# Treatment of Massive Hemoptysis with Repeated Doses of Nebulized Tranexamic Acid

**DOI:** 10.7759/cureus.29625

**Published:** 2022-09-26

**Authors:** Danica M Cutshall, Brannon L Inman, Melissa Myers

**Affiliations:** 1 Emergency Medicine, Brooke Army Medical Center, Fort Sam Houston, USA

**Keywords:** bronchial artery embolization (bae), bae, bronchoscopy, respiratory distress, nebulized, tranexamic acid, txa, hemoptysis, massive hemoptysis

## Abstract

When life-threatening hemoptysis occurs, it can be challenging to treat. We present a case of a 61-year-old female with massive hemoptysis treated with multiple doses of nebulized tranexamic acid (TXA). This treatment led to the resolution of respiratory distress and the improvement of hemoptysis. Ultimately, in cases of massive hemoptysis, repeated treatments with nebulized TXA may be a safe short-term option for symptom management prior to a more definitive therapy via bronchoscopy or bronchial arterial embolization.

## Introduction

Hemoptysis is the expectoration of blood from the lower respiratory tract, generally below the vocal cords [1]. Greater than 300cc of hemoptysis in a 24-hour period is generally agreed upon as "massive" [2]. When massive hemoptysis occurs, it carries significant mortality from 9-38% [3]. Common causes include malignancy, bronchiectasis, bronchitis, and tuberculosis [1,4]. When evaluating hemoptysis, multidetector computed tomography (CT) angiogram has largely replaced angiography as the diagnostic imaging of choice to identify bleeding arteries and other causes of respiratory distress such as pulmonary embolism.

With regards to the treatment of life-threatening massive hemoptysis, bronchoscopy (generally rigid bronchoscopy) is one of the first-line procedures for identifying bleeding in the proximal airways and providing source control of the haemorrhage [3]. Source control options include the application of cold saline, electrocautery, topical vasoconstrictors, cellulose, n-Butyl cyanoacrylate glue, and balloon tamponade [3]. Access via bronchoscopy also allows for isolation of the unaffected lung and a pathway from which to clear clots and secretions to help with respiratory distress [3]. From a consulting perspective, this procedure is generally coordinated with pulmonology.

If the culprit lesion lies beyond the proximal airways or there is evidence of diffuse airway bleeding, bronchial arterial embolization (BAE) is another treatment option. From a consulting perspective, this procedure is generally coordinated with interventional radiology. Angiography (predominately standard common femoral access) is performed first to identify abnormalities in the pulmonary artery vascular beds [2]. Generally, a bronchial artery diameter of > 3mm indicates a vascular abnormality and is a good target for microcatheter-directed embolization with polyvinyl alcohol, gelatin sponge, or coils [2].

Historically, surgery was the first-line treatment for massive hemoptysis but is now generally reserved for cases where haemorrhage is thought to be secondary to prior surgery, as the mortality rate of emergent pulmonary resection is estimated to be between 25-50% [1,3].

Tranexamic acid (TXA) is a lysine derivative antifibrinolytic often used for haemorrhage control, however, its use in pulmonary haemorrhage is underrepresented in the literature [5]. Initial case reports have shown promise in the use of single-dose nebulized TXA as a noninvasive therapeutic option for the management of massive hemoptysis, and recurrent submassive hemoptysis in cancer patients [6,7]. Additionally, one case series sought to address the use of recurrent nebulized TXA in critically ill patients [8]. However, to date, no emergency medicine-based literature has described the use of repeated doses of nebulized TXA with the intent to bridge to treatment via bronchoscopy or BAE.

## Case presentation

A 61-year-old female with a 20-pack-years smoking history, chronic obstructive pulmonary disease, and recent humeral fracture, presented to the emergency department (ED) with a chief complaint of two days of worsening small volume hemoptysis and dyspnea. The patient described the hemoptysis as streaks of blood in her sputum that had progressed to approximately 2.5-5 cc per tussive episode. Upon arrival, the patient’s vital signs were notable for a heart rate of 102 beats per minute, and SpO2 of 92% on room air, with normal blood pressure. On physical examination, the patient was in mild respiratory distress with frequent coughing episodes.

**Figure 1:**
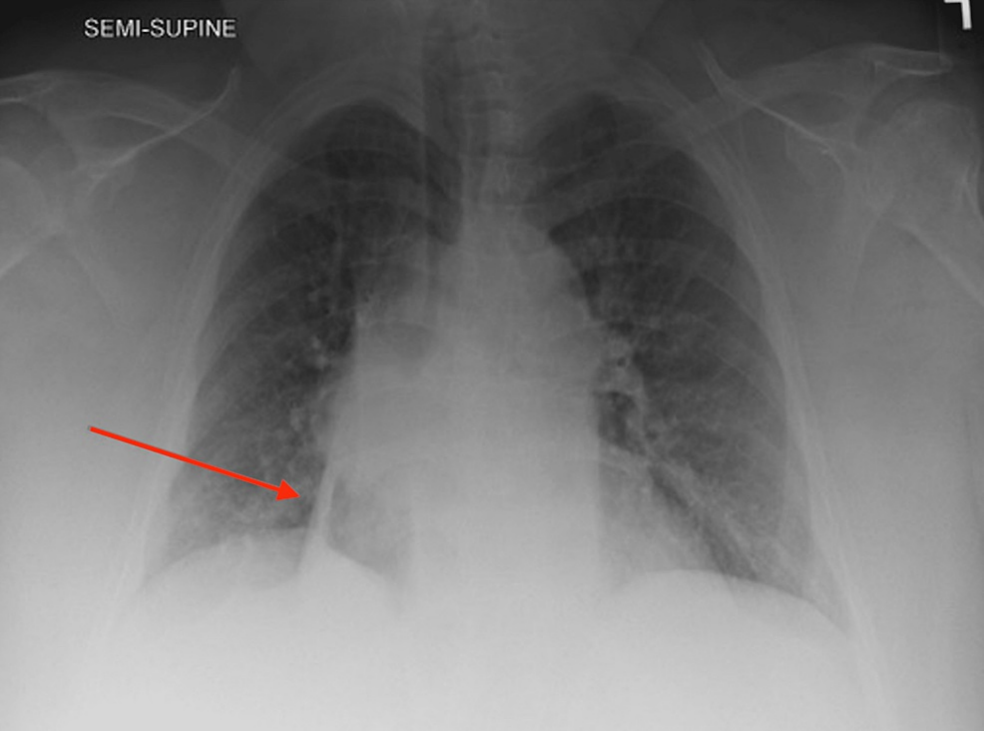
Chest X-ray revealing right lower lobe opacity concerning for lung mass (red arrow)

**Figure 2:**
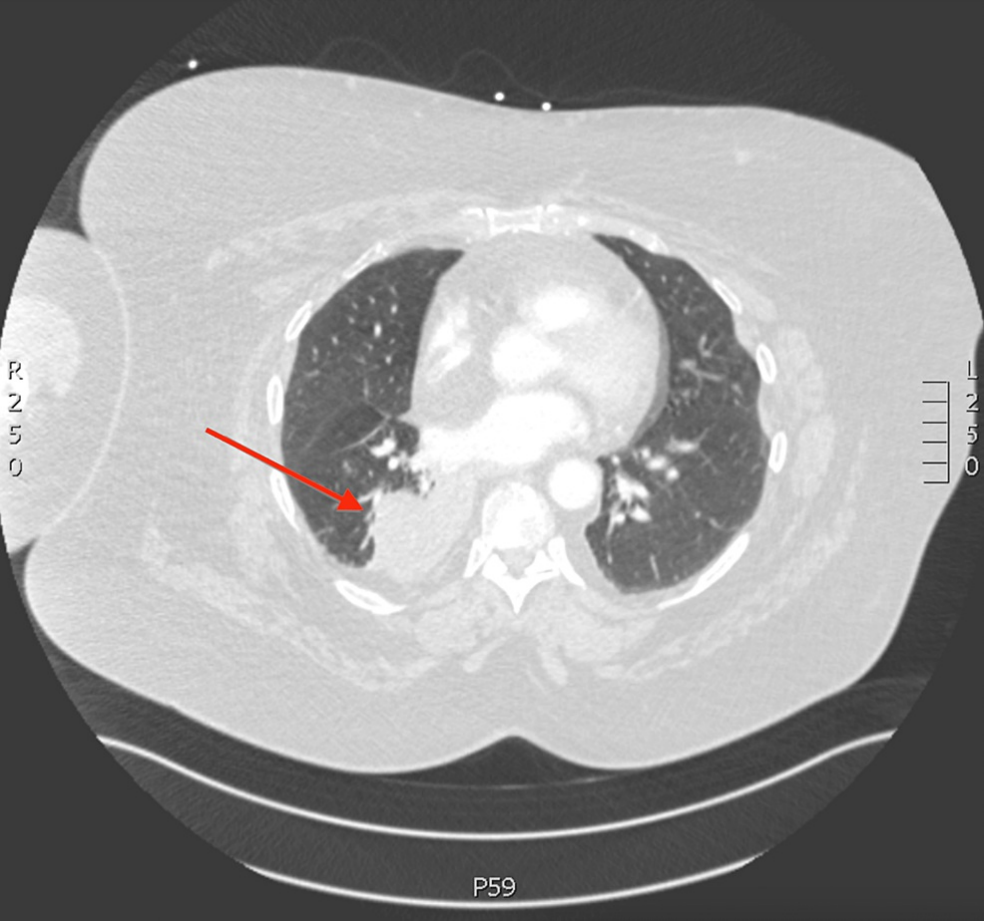
CT Chest with IV contrast revealed atelectatic lung surrounding a right peri-mediastinal mass (red arrow)

Minutes after arrival in the ED the patient had an episode of massive hemoptysis, filling a bedpan with approximately 500 cc of blood. At this time basic labs and a blood type and screen were obtained. Labs were remarkable for haemoglobin levels of 9.8 g/dL (baseline 12 g/dL), white blood cell count of 15.5x10^3^ cells/L, and a lactate of 1.5 mmol/L. The coagulation panel was normal. The chest X-ray (Figure 1) showed a right lower lung field mass, for which she was currently undergoing outpatient evaluation. Chest CT (Figure 2) showed infectious appearing atelectatic lung surrounding a right peri-mediastinal mass with peri-mediastinal and peri-tracheal lymphadenopathy. Both the mass and possible surrounding infection were considered potential sources of hemoptysis.

The patient was given 500 mg of nebulized tranexamic acid which she felt improved her symptoms and improved but did not eliminate her tussive episodes during which she had two repeat episodes of small volume hemoptysis. Broad spectrum antibiotics were started and pulmonology was consulted for definitive management. The pulmonology team planned for bronchoscopy as the definitive treatment of the patient’s hemoptysis, however, for unclear reasons, there was going to be a several-hour delay in setting up the bronchoscopy suite. Given the patient’s response to nebulized TXA, with hemodynamic stability now in the absence of respiratory distress, the recommendation was made for serial nebulized TXA treatments as a bridge to definitive therapy via delayed bronchoscopy. Following admission, three additional treatments of nebulized TXA were provided prior to her bronchoscopy with continued symptom control and no repeated bouts of massive hemoptysis. There were no documented adverse events.

## Discussion

Massive hemoptysis is a life-threatening condition that requires prompt recognition and stabilization by emergency medicine physicians. Prompt stabilization of hemoptysis may prevent the dreaded situation of needing to intubate a patient through a bloody and therefore difficult to visualize airway. Initial diagnostics of massive hemoptysis currently include chest X-rays and CT angiograms of the chest [1]. Bronchoscopy or BAE may be the definitive procedure of choice, with surgery usually limited to the management of suspected post-surgical massive hemoptysis [1,2].

Case reports have described the successful use of nebulized TXA in massive and submassive hemoptysis and pulmonary haemorrhage [6-8]. However, case reports and case series in the emergency medicine literature describe a single treatment of nebulized TXA. To date, there is a relative dearth of evidence with regards to the efficacy of multiple doses of nebulized TXA. Interestingly, the first randomized controlled trial on nebulized TXA included a 500 mg every eight hours protocol; however, this study involved treatment of non-massive hemoptysis [9].

## Conclusions

Massive hemoptysis is a life-threatening condition with the potential for airway compromise and decompensation requiring prompt treatment. Several case reports have highlighted symptom improvement after single doses of nebulized TXA. Our massive hemoptysis patient received repeated doses of nebulized TXA prior to receiving bronchoscopy. These treatments were well tolerated, with no reported adverse outcomes and resolution of her respiratory distress with improvement in hemoptysis. She proceeded to bronchoscopy where she was found to have a bleeding lesion later determined to be malignant, which was treated via electrocautery. She was discharged for further surgical planning in coordination with pulmonology and oncology.

Our case suggests that repeated treatments of nebulized TXA may be an effective therapy for symptom control during massive hemoptysis and should be further evaluated as a potential bridging therapy to more definitive treatment via bronchoscopy, bronchial arterial embolization, or in some cases, surgery.
